# **Genetic and epigenetic control of gene expression by CRISPR–Cas systems**

**DOI:** 10.12688/f1000research.11113.1

**Published:** 2017-05-25

**Authors:** Albert Lo, Lei Qi

**Affiliations:** 1 Department of Bioengineering, Stanford University, Stanford, CA 94305, USA; 2 Department of Chemical and Systems Biology, Stanford University, Stanford, CA 94305, USA; 3 ChEM-H, Stanford University, Stanford, CA 94305, USA

**Keywords:** CRISPR, Cas, dcas

## Abstract

The discovery and adaption of bacterial clustered regularly interspaced short palindromic repeats (CRISPR)–CRISPR-associated (Cas) systems has revolutionized the way researchers edit genomes. Engineering of catalytically inactivated Cas variants (nuclease-deficient or nuclease-deactivated [dCas]) combined with transcriptional repressors, activators, or epigenetic modifiers enable sequence-specific regulation of gene expression and chromatin state. These CRISPR–Cas-based technologies have contributed to the rapid development of disease models and functional genomics screening approaches, which can facilitate genetic target identification and drug discovery. In this short review, we will cover recent advances of CRISPR–dCas9 systems and their use for transcriptional repression and activation, epigenome editing, and engineered synthetic circuits for complex control of the mammalian genome.

## Introduction

Many pathological conditions such as metabolic disorders, cardiovascular diseases, cancer, or other common diseases are often attributed to dysregulated gene expression ^1– 3^. The development of methods that can accurately control gene expression contributes to our understanding of cellular physiology, which is essential for both basic biological research and the advancement of medicine. Gene expression is a multistep process that involves coordinated control of transcription, translation, and turnover of messenger RNAs (mRNA) and proteins ^4^. Precise regulation of the process by which DNA becomes RNA on the transcriptional or epigenetic level is the first step to fully control this sophisticated process.

In the past few years, RNA-guided sequence-specific genome editing and regulation technologies have been developed based on the discovery of the clustered regularly interspaced short palindromic repeats (CRISPR)–CRISPR-associated (Cas) systems. The rapid development of these tools has come after years of research on CRISPR biology, which reveals that bacteria and archaea use CRISPR-associated, RNA-guided DNA endonucleases to defend themselves from invading foreign genetic elements ^5– 17^. According to their protein sequence homology and CRISPR repeat identity, the CRISPR–Cas systems discovered so far have been categorized into two major classes and six types using bioinformatics methods ^18– 20^. The class 1 CRISPR system, which requires a large complex of several effector proteins, is divided into types I, III, and IV. In contrast, in the class 2 CRISPR system, which is divided into types II, V, and VI, only one RNA-guided endonuclease is required to mediate the cleavage of invading genetic elements ^13, 18, 21^. Due to their simplicity, the class 2 CRISPR–Cas systems have been mostly employed for genome-engineering applications. In particular, the type II CRISPR–Cas9 systems have been used in a variety of organisms including microbes ^22– 25^, fungi ^26– 29^, plants ^30– 40^, and animals ^41– 46^.

**Figure 1.**
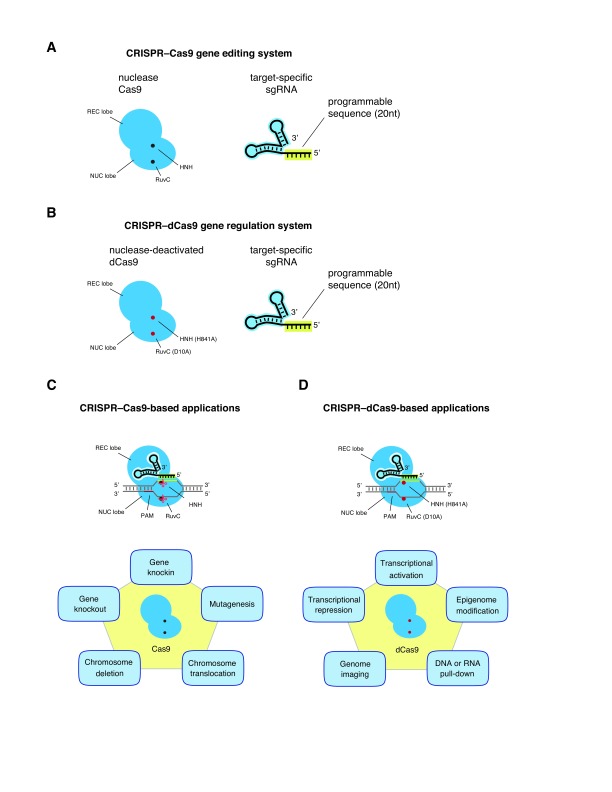
Clustered regularly interspaced short palindromic repeats (CRISPR)–CRISPR-associated 9 (Cas9) systems for genome editing and gene regulation. **A**. CRISPR–Cas9-mediated site-specific genome editing is accomplished by an RNA-guided DNA nuclease protein and a programmable single guide RNA (sgRNA). **B**. Nuclease-deficient Cas9 (dCas9) is engineered by introducing mutations (H841A and D10A) into catalytic residues of the HNH and RuvC nuclease domains. dCas9 therefore becomes a universal RNA-guided DNA-binding protein. **C**. Summary of CRISPR–Cas9-based genome engineering applications. **D**. Summary of CRISPR–dCas9-based genome engineering applications. PAM, protospacer-adjacent motif.

In the type II CRISPR–Cas systems, a Cas9 endonuclease and a guide RNA, which consists of a DNA-targeting CRISPR-associated RNA (crRNA) and the trans-activating crRNA (tracrRNA), establish a functional guide RNA–Cas9 complex ^10^. The CRISPR–Cas9 complex is recruited to the target DNA site by its guide RNA, which has a ~20 nucleotide sequence complementary to its target. The DNA target must be adjacent to a short stretch of DNA sequence termed protospacer-adjacent motif (PAM) ^47^, which is compatible with the type of Cas9 being employed. The endonuclease activity of Cas9 thus causes a double strand break (DSB) at the target site ^8^. To simplify the use of the CRISPR–Cas9 systems for genome-engineering applications, a seminal study demonstrated that crRNA and tracrRNA could be engineered into a chimeric single guide RNA (sgRNA), which is easily programmable and portable ( Figure 1A) ^6^. Adaptation of CRISPR–Cas9 systems from prokaryotes has spurred the development of genome-editing tools in eukaryotes ^41, 42, 48– 51^. Through the generation of a sequence-specific DSB by Cas9 in the host, the error-prone DNA repair pathway (non-homologous end joining) will be triggered, which often results in insertion/deletion (indel) mutations at the site of editing. Alternatively, by providing a separate DNA template containing sequences homologous to the regions flanking the DSB, Cas9 can facilitate targeted incorporation of the repair template into the genomic DNA by homology-directed repair (HDR). A summary of CRISPR–Cas9-based genome-engineering applications is provided in Figure 1C. Detailed reviews on CRISPR–Cas systems for gene editing can be found in 52– 54.

Beyond gene editing, CRISPR–Cas9 has been repurposed as a genome-docking platform that allows for a broad range of genome-engineering applications ( Figure 1B and D) ^53, 55, 56^. This is achieved by ablating the nuclease activity of Cas9 by creating mutations in the RuvC and HNH nuclease domains, which are conserved among Cas9s from various bacterial species. The resulting nuclease-deactivated Cas9 (dCas9) is a generic RNA-guided DNA-binding molecule which cannot cleave DNA but still retains its ability to bind to specific DNA sequences ^57^. dCas9 can be further engineered into programmable artificial transcription factors in eukaryotic cells to modulate gene expression when coupled with transcriptional repressors or activators, as illustrated below. The structural information about the CRISPR–Cas system has guided, and will continue to guide, the engineering efforts for applications ^53, 58^.

## CRISPR–dCas9 for transcriptional repression: CRISPR interference

**Figure 2.**
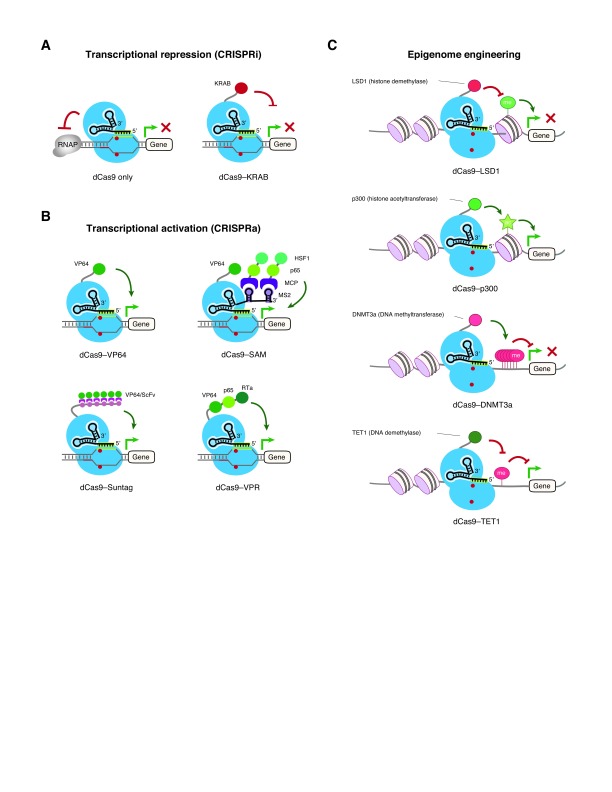
Control of gene expression by clustered regularly interspaced short palindromic repeats (CRISPR)–nuclease-deficient CRISPR-associated 9 (dCas9) systems. **A**. CRISPR interference (CRISPRi) for gene repression. The CRISPR–Cas9 complex can interfere with the assembly of RNA polymerases (RNAPs) at the transcriptional initiation step or disrupt the elongation of RNAPs to abrogate gene expression. In addition, a transcriptional repressor such as Krüppel-associated box (KRAB) can be coupled with dCas9 to achieve gene repression. **B**. CRISPR activation (CRISPRa) for gene expression. dCas9 can be tethered with a transcriptional activator, for instance: 1) four copies of the herpes simplex viral protein 16 (VP16) activation domain (dCas9–VP64); 2) an aptamer-based recruitment system termed synergistic activation mediator (SAM), which uses combinatorial RNAs including single guide RNA (sgRNA) and MS2 RNA aptamers to recruit the MS2 bacteriophage coat protein (MCP) fused with p65 and heat shock factor 1 (HSF1); 3) a SunTag system that utilizes an array of small peptide epitopes to recruit multiple copies of single-chain variable fragment (scFv) fused with VP64; 4) a tandem fusion of three transcriptional activators including VP64, p65, and the Epstein–Barr virus R transactivator (RTa), or VPR, to augment gene expression. **C**. CRISPR–dCas9-based epigenome engineering. DNA and histone landmarks can be programmed in a designer manner by coupling dCas9 with specific epigenetic modifiers. For instance, fusing LSD1 demethylase with dCas9 can erase methylation of histone H3 lysine 4 (H3K4me2) near the enhancer region to abrogate gene expression, whereas the dCas9–p300 acetyltransferase chimeric protein increases acetylation of histone H3 lysine 27 (H3K27) at the enhancer and promoter to enhance gene expression. Alternatively, the DNA methylation status at the CpG-rich promoter can be modulated by a DNA methyltransferase (DNMT3a) or demethylase (TET1) to repress or augment gene expression, respectively.

The first demonstration of dCas9-based repression was in bacteria, where it was shown that dCas9 is able to abrogate the transcription of targeted genes either by disrupting transcription factor binding or by interfering with transcriptional elongation ^57, 59– 61^ ( Figure 2A, left). As bacterial cells lack the machinery for RNA interference, this technique allows for research into gene function that was not previously possible in microbes. As CRISPR–dCas9 interferes with the transcription of the targeted genes by sterically hindering the elongation of RNA polymerase (RNAP) or inhibiting the initial binding of RNAP to the promoter, this approach is termed CRISPR interference (CRISPRi). Studies also demonstrate that CRISPRi is highly specific with minimal off-target effects in bacterial cells and allows for tunable regulation of individual genes and multiplexable control of many genes using multiple sgRNAs ^57, 60^. Exploring these unique features, CRISPRi has been applied to systematically interrogate the function of essential genes for their roles in growth, death, drug resistance, and morphology control ^62^.

Although CRISPRi using only dCas9 and an sgRNA is highly efficient and can silence gene expression by up to 99.9% in prokaryotes ^57^, it achieves only modest repression (up to 60–80%) of fluorescent reporter genes or tested endogenous genes in mammalian cells ^63, 64^. It is possible that the dCas9–sgRNA complex alone is not sufficient to fully block the action of the RNAP complex in eukaryotic cells. To improve the repression efficiency, dCas9 has been fused with a number of repressive transcriptional or epigenetic effector domains, including the KRAB (Krüppel-associated box) domain of Kox1, the CS (chromoshadow) domain of HP1α, the WPRW domain of Hes1, or four concatenated copies of the mSin3 interaction domain (SID4X) ^63, 65, 66^. Among these, KRAB–dCas9 fusion-mediated transcriptional repression is proven to be relatively robust, which can lead to downregulation of the endogenous gene in the range of 90 to 99% with a properly designed sgRNA ^63, 66^ ( Figure 2A, right).

To understand the rules for designing sgRNAs for effective transcriptional repression, high-throughput screens using 54,810 sgRNAs tiled within a 10 kilobase window near the transcription start sites (TSS) of 49 genes have been performed in K562 myelogenous leukemia cells ^66^. These experiments demonstrate that repression efficiency is strongly affected by the binding sites of the sgRNAs. In general, strong repression activity is observed by targeting the window of DNA from −50 to +300 bp relative to the TSS of a gene, with a maximum in the ∼50–100 bp region just downstream of the TSS. Moreover, using sgRNAs with a statistical spacer length of 18–21 nucleotides achieves efficient repression, whereas the targeted DNA strand and guanine-cytosine (GC) content of sgRNA are not critical factors in successful CRISPRi implementation ^66^.

## CRISPR–dCas9 for transcriptional activation: CRISPR activation

In addition to coupling with transcriptional repressors, dCas9 can be fused with a transcriptional activator to drive target gene expression ( Figure 2B). This CRISPR–dCas9-based gene activation is termed CRISPR activation (CRISPRa). In bacteria, fusion of the ω subunit of RNAP to the dCas9 can upregulate reporter gene expression up to 3-fold in *Escherichia coli* ^59^. Further tool development is needed for the practical implementation of CRISPRa in bacterial cells. For instance, characterization of additional transcriptional activators that function in bacterial cells is necessary for enhanced activation efficiency. Furthermore, testing how a given transcriptional activator functions in broad species of bacteria will be important to understand the true utility of the method in the kingdom of microbes.

In contrast to those in microbes, more CRISPRa tools have been developed for mammalian cells. dCas9 has been fused to various transcriptional activators, including the transactivation domain of NF-κB p65 subunit (p65AD), the Herpes simplex viral protein 16 (VP16), or multiple tandem copies of VP16, such as VP64 (four copies, Figure 2B) or VP160 (10 copies) ^67, 68^. While these simple fusion proteins are able to activate the expression of reporters or endogenous genes, the potency of these constructs in transcriptional activation is quite modest, about 2-fold to 5-fold on average when using a single sgRNA ^63, 67, 69– 71^. The effectiveness of CRISPRa can be increased by using multiple sgRNAs tiled across the promoter of the target gene, which is tedious and sometimes challenging, as it requires co-delivery of many sgRNAs simultaneously ^67, 70^.

Several attempts have been made to improve the efficiency of CRISPRa. One approach is to amplify the activation signal from the transcriptional activator VP64 ^66, 72^. This is achieved by fusing a scaffold to dCas9 that is able to recruit many copies of VP64. This scaffold consists of a tandem array of antibody epitopes, named SunTag array, which can specifically interact and recruit multiples copies of a single-chain variable fragment (scFv) fused to VP64 ( Figure 2B) ^66, 72^. This system can significantly increase the expression of endogenous genes, e.g. CXCR4, up to 50-fold in human erythroleukemia K562 cells with a single sgRNA as compared to a 2-fold increase observed with dCas9–VP64 fusion ^73^. Using dCas9–SunTag, potent activation of CXCR4 is shown to promote cell migration ^72^.

Another approach to augment transcriptional activation is by tethering multiple different activators to the dCas9. Researchers have fused dCas9 with a tripartite transactivator composed of VP64, p65AD, and Epstein–Barr virus R transactivator (Rta) (VPR) in tandem ( Figure 2B) ^74^. The dCas9–VPR system exhibits improved activation of endogenous coding and non-coding genes using multiple sgRNAs when compared with dCas9–VP64 fusion. This system is shown to direct the neuronal differentiation of induced pluripotent stem cells by using multiple sgRNAs against each target gene ^74^.

A third approach is to use sgRNAs as scaffolds to recruit multiple transactivators that can function synergistically in enhancing the activation of endogenous genes. This method is referred to as the synergistic activation mediator (SAM) system ( Figure 2B) ^75^. This system is composed of a modified sgRNA containing two copies of an MS2 RNA hairpin (from the MS2 bacteriophage). Each MS2 hairpin recruits a pair of its cognate RNA-binding protein MS2 bacteriophage coat protein (MCP), which is fused with p65AD and heat shock factor 1 (HSF1). When combined with dCas9–VP64, the SAM system is shown to robustly activate endogenous coding and non-coding genes and has been employed for genome-wide screening for genes that confer resistance to a BRAF inhibitor ^75^.

While these CRISPRa systems all exhibit significant enhancement in terms of target gene activation efficiency compared to dCas9–VP64 fusion, systemic comparisons reveal their ability to robustly activate gene expression in a range between 10-– and 1,000-fold depending on the specific gene being perturbed ^76^. Moreover, these systems function effectively in a variety of mammalian cell types, including human, mouse, and *Drosophila*. Attempts to further enhance efficiency by combining elements from VPR, SAM, and SunTag have been unsuccessful, suggesting that our understanding of the synthetic activation of endogenous genes in the genome is still limited ^76^. Next-generation CRISPRa systems should incorporate novel activation domains, very likely by combining them with synergistic epigenetic modifiers to drive highly effective gene activation.

## CRISPR–dCas9 for epigenome editing

Epigenetic modifications of the genome provide an important mechanism for heritable gene expression ^77– 81^. Modifications of the chromatin DNA or associated histones are correlated with distinct transcription states and disease phenotypes. For instance, promoters with silenced transcription exhibit distinct methylation or acetylation marks (e.g. histone H3 lysine 9 [H3K9] methylation, histone H3 lysine 27 [H3K27] methylation, and/or DNA methylation), while promoters with active transcription are associated with other unique patterns of modifications (e.g. histone acetylation or histone H3 lysine 4 [H3K4] methylation). Tools that allow us to precisely modify epigenetic marks at a given locus will be critical for understanding the basic and translational epigenetics. Since CRISPR–dCas9 allows for highly specific genomic targeting, its site-specific recruitment of chromatin-modifying epigenetic enzymes has been tested for sequence-specific epigenome editing ( Figure 2C).

By coupling histone demethylase LSD1 with dCas9, this fusion protein is able to effectively repress pluripotency maintenance genes (e.g. Oct4 and Tbx3) by using sgRNAs to target the enhancer regions of these genes in mouse embryonic stem cells ^82^. Decreased expression of target genes is correlated with reduced levels of H3K4 dimethylation (H3K4me2) and H3K27 acetylation (H3K27ac) near the enhancer regions. Loss of expression of these pluripotency maintenance genes eventually causes morphological changes in embryonic stem cells ^82^. Interestingly, the dCas9–KRAB fusion protein can also induce the repressive trimethylation of H3K9 (H3K9me3) when targeted to the HS2 enhancer of the human beta-globin locus, thereby inhibiting the expression of globin genes ^83^.

For activating epigenetic modifications, the catalytic domain of the human acetyltransferase p300 has been fused to dCas9, which is able to increase the level of H3K27ac at the enhancer and promoter of targeted genes, resulting in their transcriptional activation ^84^. Furthermore, integrating the catalytic domain of histone methyltransferase PRDM9 with dCas9 or zinc finger (ZF) proteins has been shown to increase the level of trimethylated H3K4 (H3K4me3) and reactivates silenced target genes in mammalian cells ^85^. The maintenance of the reactivated state, however, is strongly dependent on the DNA methylation status of the CpG islands at their promoters. For example, gene re-expression achieved by targeted epigenetic editing can be maintained in DNA hypomethylated loci (e.g. PLOD2 in C33a cells), in contrast to transient reactivation observed in hypermethylated loci (e.g. EpCAM in HeLa cells). Stable gene reactivation depends on the presence of methylation marks on histone H3 lysine 79 (H3K79me), which is required for the stability and maintenance of H3K4me3. Epigenetic editing to enrich H3K4me3 by dCas9–PRDM9 and H3K79me by dCas9–DOT1L is able to sustain gene reactivation in a hypermethylated locus when DNA methyltransferase is inhibited ^85^.

Gene expression can also be modulated by dCas9-based regulation of DNA methylation. Fusion of the catalytic domain of the DNA demethylase TET1 to dCas9 can induce targeted DNA demethylation ^86– 89^. The dCas9–TET1 fusion can demethylate 30 to 60% of the CpG islands at tested promoters, which drives the transcriptional activation of target genes in various cell types including embryonic stem cells, cancer cell lines, fibroblasts, and primary neurons ^86– 89^. Alternatively, tethering of the full-length or catalytic domain of DNA methyltransferase 3A (DNMT3A) to dCas9 is shown to induce methylation of the CpG islands at a range of 30 to 50% of the sgRNA-targeted genomic loci, thus leading to reduced expression of those targeted genes in various cell types ^86, 90, 91^. Interestingly, triple recruitment of DNMT3A, DNMT3L, and KRAB via different fusion constructs is able to achieve up to 100% of methylation of the CpG islands and results in stable loss of expression of the targeted genes ^92^. Gene silencing mediated by co-recruitment of such epigenetic modifiers is resistant to external transcriptional activation stimuli, for instance dCas9–VP160 or dCas9–p300, but can be reversed by targeted DNA demethylation mediated by dCas9–TET1 ^92^.

Together, these studies have demonstrated how to utilize dCas9-based epigenetic modifiers for locus-specific epigenetic editing in mammalian cells. In the future, it will be intriguing to investigate how epigenetic modifications alter local genomic structure. Meanwhile, further expanding and combining novel epigenetic modifier tools may serve as the key to improve regulatory controls of endogenous gene expression. The dCas9 epigenetic editing toolkit will become a key component for understanding the relationship between chromatin states, gene regulation, and cellular phenotypes.

## Multimodal function and multi-dimensional controls of CRISPR–(d)Cas9

**Figure 3.**
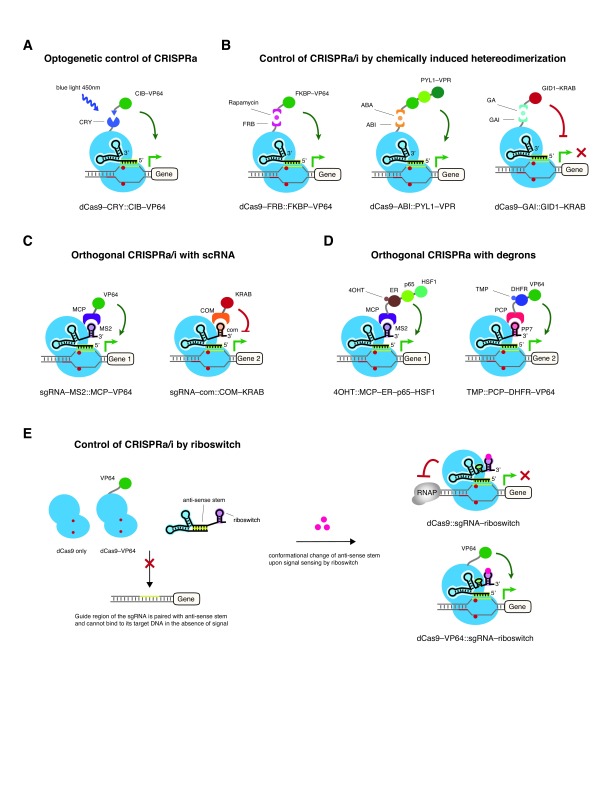
Synthetic approaches for complex and precise control of clustered regularly interspaced short palindromic repeats (CRISPR)–nuclease-deficient CRISPR-associated 9 (dCas9) systems. **A**. Control of CRISPR–dCas9 systems by light-inducible heterodimerization proteins. dCas9 and transcriptional effector (e.g. four copies of Herpes simplex viral protein 16 [VP64]) can be decoupled and fused to CRY and CIB, respectively. Upon sensing blue light, VP64 is recruited to dCas9 to activate gene expression. **B**. CRISPR–dCas9 systems can be rendered chemically controlled. Small-molecule-induced heterodimerization domains (e.g. FRB::FKBP, ABI::PYL1, and GAI::GID1) are introduced to dCas9–effector split systems for transcriptional controls. Binding of corresponding chemical (e.g. rapamycin, abscisic acid [ABA], or gibberellin [GA]) induces gene regulation by CRISPR–dCas9. **C**. Simultaneous gene activation and repression using a scaffold RNA (scRNA) system. A hybrid RNA consisting of an sgRNA and an RNA aptamer (e.g. MS2, com, PP7) is able to recruit the cognate RNA-binding protein (e.g. MCP, COM, PCP) tethered to either an activator (e.g. VP64) or a repressor (e.g. Krüppel-associated box [KRAB]). **D**. CRISPR–dCas9 systems can be regulated by protein destabilization domains. Structurally unstable protein domains such as estrogen receptor (ER) or the dihydrofolate reductase (DHFR) are fused to a chimeric protein consisting of an aptamer-binding protein (e.g. MCP or PCP) and a transcriptional activator (e.g. p65–HSF1 or VP64). These unfolded and destabilized domains increase fusion protein turnover by rapid proteasome-mediated degradation. Small molecules can bind and stabilize those domains (e.g. binding of 4OHT [4-hydroxytamoxifen] stabilizes ER, and binding of TMP [trimethoprim] stabilizes DHFR), allowing the control of gene expression. **E**. Control of CRISPR–dCas9 systems by RNA switchable devices. The single guide RNA (sgRNA) can be engineered to become a biosensor by fusing it with signal-responsive RNA aptamers. In the absence of certain signals, the guide region of the sgRNA pairs with the antisense stem and therefore cannot bind to its target DNA. Upon sensing specific signals by the RNA aptamers, a conformational change allows the guide region of the sgRNA to bring the dCas9 or dCas9–effector complex to the target DNA site, thereby altering gene expression.

One of CRISPR–dCas9’s unique strengths is its ability to regulate the expression of multiple genes in a simultaneous and inducible manner. Novel regulatory approaches that combine Cas9 or dCas9 with optogenetics, chemical biology, RNA, and protein engineering have been created to facilitate the dynamic and spatiotemporal control of target genes ( Figure 3).

For instance, an optogenetic transcriptional control system can be integrated into CRISPR–dCas9 ^93, 94^. By decoupling dCas9 from its transcriptional effector (e.g. VP64 or p65AD) and fusing each member to a light-inducible heterodimerizing pair of proteins (e.g. CRY–CIB system), effectors can be recruited to dCas9 to activate endogenous genes in a rapid and reversible manner upon exposure to blue light ( Figure 3A). Furthermore, CRISPR–dCas9 can be integrated with small-molecule-mediated transcriptional systems to modulate the expression of targeted genes ^95^. One system uses dCas9–VP64 split into two components, which are then fused to rapamycin-binding dimerization domains FK506 binding protein 12 (FKBP) and FKBP rapamycin binding (FRB) ( Figure 3B). In the presence of rapamycin, dCas9–VP64 is reassembled and can activate the transcription of multiple genes to a level comparable to that of the full-length dCas9–VP64 in HEK293FT cells. However, transcriptional activation of these genes is irreversible upon withdrawal of rapamycin ^95^. In another study, direct evolution of Cas9 using randomized insertional mutagenesis revealed hotspots for incorporating small synthetic protein domains into Cas9 without compromising the enzyme's RNA-guided DNA-binding activity. Insertion of the ligand-binding domain of human estrogen receptor-α renders dCas9 allosterically responsive to 4-hydroxytamoxifen (4OHT), thereby permitting the precise regulation of CRISPRi activity by this drug ^96^.

In order to enable simultaneous editing and/or control of multiple genes within the same cell population, Cas9 orthologs have been characterized and employed ^97– 99^. For instance, Cas9 from *Streptococcus pyogenes* and *Neisseria meningitidis* displays distinct patterns in its recognition of target sequences and therefore can mediate independent transcriptional repression and nuclease activity ^97^. Furthermore, orthogonal dCas9 regulators that utilize multiple chemically induced dimerization systems have been developed to allow the temporal control of gene regulation ^100, 101^. In a screen of chemical- and light-inducible heterodimerization systems to control the association of dCas9 with effectors, the potent chemical inducers abscisic acid (ABA) and gibberellin (GA) were shown to mediate efficient gene activation and repression in mammalian cells ^100^ ( Figure 3B). Fusion of ABA and GA inducer systems with orthogonal dCas9 regulators can independently control the expression of different genes within the same cell. Moreover, these systems can be used to devise basic Boolean logic-gated dCas9 operators to achieve orthogonal and multiplexed transcriptional modulation ^100^. Separately, another study reported CRISPR–dCas9 activators that can be chemically induced with rapamycin and GA-mediated dimerization systems to activate endogenous human genes ^101^.

In addition to its use in protein engineering, the sgRNA can also be engineered as a scaffold RNA (scRNA) to expand the utility of CRISPR–dCas9 systems ( Figure 3C). The scRNA, created by appending protein-binding RNA aptamers to the sgRNA, can serve as an adaptor to recruit the cognate RNA-binding proteins (RBPs) that fused with the transcriptional repressor or activator ^73, 102^. This design allows for simultaneous gene activation and repression within the same cell when orthogonal scRNA aptamer–RBP pairs are used for different gene targets. Furthermore, RBPs can be modified with small-molecule-mediated protein degradation domains (degrons) to offer fine-tuned control of the targeted gene’s regulation ^103^ ( Figure 3D). Additionally, by coupling riboswitches (i.e. switchable RNA devices that recognize specific stimuli) with sgRNA, the dCas9–sgRNA complex is able to function as a type of “signal conductor” that can regulate the transcription of endogenous genes in response to external or internal riboswitch-responsive signals ^104^ ( Figure 3E). dCas9-based signal conductors can also be applied for rewiring signaling pathways by devising artificial links that couple different signaling components. These systems can also be used to reprogram the fate of cancer cells by converging oncogenic signaling into an anti-oncogenic pathway ^104^. More sophisticated circuits with layered regulatory mechanisms have been designed to control Cas9-based transcriptional repression machinery in mammalian cells ^105^.

Approaches such as using split Cas9 variants ^95, 106– 108^, transient delivery of Cas9:sgRNA ribonucleotide protein complexes (RNPs) ^109– 111^, engineered allosteric protein or RNA switches, and novel optogenetic or chemogenetic systems have also been applied to the CRISPR–Cas9 method for gene editing for the purpose of generating conditional gene knockouts and for reducing the levels of off-target effects ^95, 96, 112– 119^. Expanding these approaches to the CRISPR–dCas9 systems will likely result in new features of regulation for dCas9-based control of gene expression.

## CRISPR–dCas9 systems versus previous existing gene regulation techniques

There are several existing methods for gene repression, gene activation, and epigenome modification. For example, techniques based on RNA interference (RNAi), which consists of small interfering RNAs (siRNA) or short hairpin RNAs (shRNA), allow for sequence-specific repression of endogenous genes of interest in eukaryotic organisms. This gene silencing effect is mediated by transcript-specific degradation due to Watson–Crick base-pairing between mRNA and siRNA or shRNA (for reviews, see 120– 123). On the other hand, the overexpression of genes of interest can be achieved by cloning open reading frames (ORFs) or cDNAs followed by vector-mediated gene transfer ^124^. A number of genome-targeting technologies such as ZF proteins ^125– 128^ or transcription activator-like effector nucleases (TALENs) ^128– 135^ have been devised for gene-editing applications, which are achieved by fusing endonuclease catalytic domains with modular DNA-binding proteins to induce DSBs at the targeted genomic loci. Targeting strategies based on protein–DNA interaction using ZF and TALENs can also be applied for gene regulation or epigenome medication when coupled to the transcriptional repressor, activator, or epigenetic modifiers mentioned earlier. Compared to these targeting techniques, CRISPR–dCas9-based gene regulation systems are easier to design, highly specific and efficient, cost effective, and well-suited for high-throughput and multiplexed gene regulation across many cell types and organisms. Nevertheless, limitations do exist for CRISPR–dCas9-based gene regulation systems depending on the application of interest. For instance, RNAi-mediated gene silencing and/or ORF overexpression techniques can be applied to study the function of gene splice variants. Comparisons of CRISPRi/a versus existing common gene repression or activation systems are summarized in Table 1 and Table 2.

**Table 1.** Comparison of CRISPRi to other gene repression techniques.

Repression technique	Design principle	Engineering	Cost	Mechanism of action	Limitation in choosing target region	Off-target effects	Ability to target splice variants	Ability to target non-coding RNAs	Capacity for genome- scale screen	Refs
**CRISPRi**	Programmable ~20 nt sgRNA to pair with the target DNA; dCas9 itself (prokaryote) or fusion with a repressor (eukaryote)	Simple	Low	Inhibition of transcriptional initiation or elongation	PAM adjacent to a target sequence	Minimal	Yes, if variants have different TSS	Yes	Yes	5, 6, 57, 59, 60, 63, 71, 73, 136
**RNAi**	Programmable ~21 nt RNA to pair with the target RNA	Simple	Low	The target RNA transcript is degraded or sequestered	No restriction	Can be extensive owing to sequence complementarity to the siRNA seed region in multiple transcripts	Yes	Yes	Yes	Reviewed in 120– 123
**ZF**	Composite of ZF motifs that binds to ~3–6 nt triplets of the target DNA; fusion ZF with a repressor	Challenging	High	Inhibition of transcriptional initiation or elongation	Confined to sequences composed of triplets with corresponding ZF	Minimal	Yes, if variants have different TSS	Yes	No	Reviewed in 137, 138
**TALE**	Composite of a series of repeat variable domains that binds to a single nt in the target DNA; fusion TALE with a repressor	Challenging	High	Inhibition of transcriptional initiation or elongation	Target must start with a T	Minimal	Yes, if variants have different TSS	Yes	No	139– 141

CRISPR, clustered regularly interspaced short palindromic repeats; CRISPRi, CRISPR interference; dCas9, nuclease-deficient CRISPR-associated 9; nt, nucleotide; PAM, protospacer-adjacent motif; RNAi, RNA interference; sgRNA, single guide RNA; siRNA, small interfering RNA; TALE, transcription activator-like effector; TSS, transcription start site; ZF, zinc finger.

**Table 2.** Comparison of CRISPRa to other gene activation techniques.

Activation technique	Design principle	Engineering	Cost	Gene activated	Limitation in choosing target region	Off-target effects	Ability to overexpress splice variants or mutant	Blocked by DNA methylation	Capacity for genome- scale screen	Refs
**CRISPRa**	Programmable ~20 nt sgRNA to pair with the target DNA; dCas9 fusion with an activator	Simple	Low	Endogenous	PAM adjacent to a target sequence	Minimal	Yes, if variants have different TSS; not ideal for expressing mutant alleles	No	Yes	6, 63, 66– 75
**ORF transfer**	Target cloning followed by gene transfer	Simple –Medium	Low	Exogenous	High GC content and long targets can be difficult to clone	None	Yes	N/A	Yes (biases may exist for larger ORFs or certain splice variants)	Reviewed in 124
**ZF**	Composite of ZF motifs that binds to ~3–6 nt triplets of the target DNA; fusion ZF with an activator	Challenging	High	Endogenous	Confined to sequences composed of triplets with corresponding ZF	Minimal	Yes, if variants have different TSS; not ideal for expressing mutant alleles	Yes	No	Reviewed in 137, 138
**TALE**	Composite of a series of repeat variable domains that binds to a single nt in the target DNA; fusion TALE with an activator	Challenging	High	Endogenous	Target must start with a T	Minimal	Yes, if variants have different TSS; not ideal for expressing mutant alleles	Yes, but can also recognize specifically	No	74, 84, 130, 133, 134, 142, 143

CRISPR, clustered regularly interspaced short palindromic repeats; CRISPRa, CRISPR activation; dCas9, nuclease-deficient CRISPR-associated 9; GC, guanine-cytosine; nt, nucleotide; ORF, open reading frame; PAM, protospacer-adjacent motif; sgRNA, single guide RNA; TALE, transcription activator-like effector; TSS, transcription start site; ZF, zinc finger.

## Conclusions

The discovery of CRISPR–Cas as an RNA-guided DNA endonuclease system has inspired the development of revolutionary gene editing tools that allow for studying the function of the genome on a cellular and organismic level. In addition to the complete loss-of-function perturbations made possible by nuclease Cas9, the development of dCas9-based CRISPRi and CRISPRa technologies has enabled reversible, multiplexed, and loss- and/or gain-of-function studies of genes of interest. Furthermore, the ability to site-specifically target chromatin modifiers fused to dCas9 will contribute to our understanding of epigenomic regulation. Genome-wide screening based on CRISPR–dCas9 systems are helping to untangle complex biological processes and identify key genetic players of diseases ^66, 75^. Integrating the dCas9 transcriptional or epigenetic tools with tools based on Cas9 gene editing, RNAi tools on the RNA level, and other molecular tools will undoubtedly constitute the next-generation toolkit for controlling inheritance and the central dogma. We imagine the diversity, plasticity, and flexibility of CRISPR–dCas9 systems will allow researchers to develop more advanced genome-engineering tools for a wide range of applications in basic research and translational medicine.
